# Quality of life and religious-spiritual coping in palliative cancer care patients

**DOI:** 10.1590/1518-8345.1857.2910

**Published:** 2017-07-10

**Authors:** Ticiane Dionizio de Sousa Matos, Silmara Meneguin, Maria de Lourdes da Silva Ferreira, Helio Amante Miot

**Affiliations:** 1 RN. Scholarship holder at Fundação de Amparo à Pesquisa do Estado de São Paulo (FAPESP), Brazil.; 2 PhD, Assistant Professor, Faculdade de Medicina de Botucatu, Universidade Estadual Paulista Júlio de Mesquita Filho, Botucatu, SP, Brazil.; 3 PhD, Adjunct Professor, Faculdade de Medicina de Botucatu, Universidade Estadual Paulista Júlio de Mesquita Filho, Botucatu, SP, Brazil.

**Keywords:** Palliative Care, Quality of Life, Spirituality, Religion, Nursing, Care Humanization

## Abstract

**Objectives:**

to compare the quality of life and religious-spiritual coping of palliative cancer care patients with a group of healthy participants; assess whether the perceived quality of life is associated with the religious-spiritual coping strategies; identify the clinical and sociodemographic variables related to quality of life and religious-spiritual coping.

**Method:**

cross-sectional study involving 96 palliative outpatient care patient at a public hospital in the interior of the state of São Paulo and 96 healthy volunteers, using a sociodemographic questionnaire, the McGill Quality of Life Questionnaire and the Brief Religious-Spiritual Coping scale.

**Results:**

192 participants were interviewed who presented good quality of life and high use of Religious-Spiritual Coping. Greater use of negative Religious-Spiritual Coping was found in Group A, as well as lesser physical and psychological wellbeing and quality of life. An association was observed between quality of life scores and Religious-Spiritual Coping (p<0.01) in both groups. Male sex, Catholic religion and the Brief Religious-Spiritual Coping score independently influenced the quality of life scores (p<0.01).

**Conclusion:**

both groups presented high quality of life and Religious-Spiritual Coping scores. Male participants who were active Catholics with higher Religious-Spiritual Coping scores presented a better perceived quality of life, suggesting that this coping strategy can be stimulated in palliative care patients.

## Introduction

In Brazil, palliative care is an emerging end-of-life care modality that has gained emphasis in recent years due to the increased life expectancy of the population, the change in the epidemiological profile of chronic-degenerative diseases and the need to provide a dignified death to patients whose illness no longer responds to the curative treatment^1^.

This fact has compelled the health professionals to rethink the way they take care of patients beyond possibilities of cure, in view of countless difficulties at home, contributing to the institutionalization of death.

Care in the palliative care context differs from curative care because it reaffirms life and faces death as a reality to be experienced together with the family members. Its purpose is to improve the patients and relatives’ Quality of Life (QoL) in view of an advanced disease, through the prevention and relief of suffering, pain treatment and valuation of the culture, spirituality, customs and values, besides the desires and beliefs that permeate death^2^ ^-^ ^3^.

Both cancer and its treatment can negatively influence the perceived QoL. Therefore, its assessment is considered a critical measure in oncology. Nevertheless, when cure and the extension of life are no longer possible, this measure becomes fundamental.

The discussions about QoL among health professionals and patients are frequent but, often, the control of physical symptoms is emphasized, while little attention is paid to the psychological, social and spiritual aspects^4^.

Religion and spirituality are constructs adopted to cope with the stress the cancer causes as, for many patients, they can contribute to the relief of suffering and greater hope concerning the QoL^5^.

Although distinct, both are intertwined, as spirituality is considered to be the essence of a person, as if it were a search for meaning and purpose in life, while religiosity is the expression of spirituality itself, through rituals, dogmas and doctrines^6^ ^-^ ^7^.

In that context, religious coping refers to the use of faith, religion or spirituality in coping with stressful situations or crisis moments, which happen in the course of life. Therefore, its study should be broad and based on a functional view of religion and the role it plays in coping^8^.

Although the religious coping concept has a positive bias, it can be both positive and negative, and the same is true for its strategies. The positive aspect combines measures that offer beneficial effects to individuals, while the negative aspect is related to the measures that entail harmful consequences, such as questioning their existence, delegating the solution of problems to God, defining stress as a punishment from God, among others^8^ ^-^ ^9^.

The relations between religiosity and palliative care have been increasingly investigated and evidence appoints a relationship that is positive in most cases. Studies demonstrate that religiosity and spirituality improve the Religious-Spiritual Coping (RSC) and QoL, besides contributing to reduce the remission time of depression^10^ ^-^ ^13^. Nevertheless, the relation between QoL and RSC in palliative care has been hardly discussed in the literature, despite the importance of this theme.

The research hypothesis is that the perceived QoL and RSC are influenced by religion/spirituality, as well as by the patients’ sociodemographic and clinical variables.

In view of the lack of studies, this research was proposed to answer the following questions.

-What is the quality of life of palliative care patients?

-Do palliative care patients use religious-spiritual coping? How?

-Is there a difference between the perceived quality of life and religious-spiritual coping in palliative care patients and a group of healthy participants?

-Is the perceived quality of life related with the religious-spiritual coping of palliative cancer care patients?

-Is there a difference between quality of life and religious-spiritual coping according to the clinical and demographic variables?

In view of the above, the objectives in this study were to compare the QoL and RSC of palliative care patients with a group of healthy participants, to assess whether the perceived quality of life is associated with the religious-spiritual coping strategies of palliative care patients and to identify the clinical and sociodemographic variables related to QoL and RSC.

## Methods

An exploratory, cross-sectional and comparative study with a quantitative approach was undertaken. The study was developed at a palliative outpatient clinic of a public hospital in the interior of the state of São Paulo between March 1^st^ 2015 and February 29^th^ 2016.

To test the study hypotheses, the participants were divided in two groups, being: Group A (case), including palliative care patients, and Group B (control) with healthy participants.

Male and female patients who complied with the following inclusion criteria were considered eligible for the study: age 18 years or older, under outpatient monitoring, in self-referred emotional conditions to answer the questionnaire and agreeing to participate in the research. Family members who did not conclude the completion of the data collection instrument were excluded.

The control group consisted of parents of healthy undergraduate nursing students from Botucatu Medical School (FMB-Unesp). Patients with chronic, mental, degenerative and progressive conditions were excluded.

To collect the data, four instruments were used. The first consisted of sociodemographic data, collected during the application of the questionnaire, and the second was the Portuguese version of the McGill Quality of Life Questionnaire (MQOL^)(^ ^14^.

It should be clarified that few specific questionnaires exist to assess palliative care patients’ quality of life. Among these, the MQOL presents the largest number of validations in other languages and higher psychometric property measures. This questionnaire consists of 16 questions in five subscales to assess palliative care patients’ quality of life: physical wellbeing, psychological wellbeing, existential wellbeing, support and physical symptoms. In addition, an additional item (Part A) measures the global quality of life and is not used for the sake of comparison with the total MQOL. This questionnaire also contains an open-ended question for the patients to describe what items most strongly influenced their quality of life. The total MQOL score corresponds to the average of the five subscales and is classified as worse the closer it is to 0, and better the closer it gets to 10^14^.

The third instrument served to assess the use of the Brief Religious-Spiritual Coping scale (CRE-Breve). The CRE scale is a North-American tool that contains 92 items, originally called RCOPE^15^, whose short version has been validated for the Brazilian culture^16^. The CRE-Breve contains 49 items, divided in two main dimensions: Positive RSC (transformation of one’s self and/or one’s life; actions in search of spiritual help; offering help to the other; positive position towards God; actions in search of the institutional other; personal search for spiritual knowledge; distancing through God, religion and/or spiritual aspects) and Negative RSC (negative revaluation of God; negative position towards God; negative revaluation of the meaning; dissatisfaction with the institutional other). The answers vary from 1 to 5 on a Likert-style scale. In total, scores between 1.0 and 1.5 correspond to none or negligible; between 1.51 and 2.50 low; between 2.51 and 3.50 average; between 3.51 and 4.50 high and between 4.51 and 5.0 very high^16^.

In this research, direct kinship was considered as the relationship in which people are blood-related, while indirect kinship is considered as marriage-related. Each participant answered the questionnaire in a private room, individually and, if the questionnaire could not be answered, an appointment was made at each relative’s convenience. In addition, it was informed that the refusal to participate in the study would imply no losses of any kind for the continuity of care.

As little knowledge exists on the QoL and RSC indicators in this population, for a 20% effect size and 95% reliability, the minimum sample size was estimated as 96 patients for each group.

Initially, all variables were analyzed descriptively. The proportions between the groups were compared using Pearson’s chi-squared or the chi-squared trend test, and quantitative data were compared by means of the Mann-Whitney test. The intergroup comparison of the median RSC-Brief and QoL scores was executed using the Mann-Whitney test. Spearman’s correlation coefficient and its respective significance tests were applied to explore the correlation among the variables. The variation in the RSC-Brief and QoL scores was evaluated in relation to the clinical and demographic variables and RSC by means of a generalized linear model (gamma probability distribution and identity link function). The analyses were developed in IBM, SPSS, version 22. Significance was set at 5%.

Approval for the study was obtained from the Ethics Committee at Botucatu Medical School under Opinion No. 969503.

## Results

Based on the inclusion criteria, 192 subjects were selected for the study sample, being 96 in each group. In Table 1, the participants’ sociodemographic characteristics are displayed. Female subjects with a partner who were active Catholics prevailed in both groups. The participants in Group A were significantly older, with lower education levels, mostly living alone and practicing religion more frequently.

**Table 1:** Sociodemographic characteristics of research participants. Botucatu, SP, Brazil, 2015

**Variable**		**Group**		
		**A** **N (%)**	**B** **N (%)**	**Total** **N (%)**
**Age (years)**				
	**Median**	63*	**41**	**53**
	**(p25-p75)**	**(53.3-70.0)**	**(26.3-52.5)**	**(35.3-64.0)**
**Sex**				
	**Male**	**38 (39.6)**	**34 (35.4)**	**72 (37.5)**
	**Female**	**58 (60.4)**	**62 (64.6)**	**120 (62.5)**
**Marital status**				
	**With partner**	**59 (61.5)**	**68 (70.8)**	**127 (66.1)**
	**Without partner**	**37 (38.5)**	**28 (29.2)**	**65 (33.9)**
**Religion**				
	**Catholic**	**67 (69.8)**	**66 (68.8)**	**133 (69.3)**
	**Non Catholics**	**29 (30.2)**	**30 (31.3)**	**59 (30.7)**
**Actively religious**				
	**Yes**	79 (82.3)*	**62 (64.6)**	**141 (73.4)**
	**No**	**17 (17.7)**	**34 (35.4)**	**51 (26.6)**
**Whom they live with**				
	**Direct kinship**	55 (57.3)*	**72 (75)**	**127 (66.1)**
	**Indirect kinship**	**29 (30.2)**	**16 (16.7)**	**45 (23.4)**
	**Alone**	**12 (12.5)**	**8 (8.3)**	**20 (10.4)**
**Education**				
	**Primary**	61 (63.5)*	**20 (20.8)**	**81 (42.2)**
	**Secondary**	**26 (27.1)**	**35 (36.4)**	**61 (31.8)**
	**Higher**	**9 (9.4)**	**41 (42.7)**	**50 (26)**
**Family income (minimum wages)**				
	**Less than one**	**3 (3.1)**	**4 (4.2)**	**7 (3.6)**
	**Between 1 and 3**	**61 (63.5)**	**40 (41.7)**	**101 (52.6)**
	**Between 4 and 10**	**31 (32.3)**	**43 (44.8)**	**74 (38.5)**
	**More than 10**	**1 (1.0)**	**9 (9.4)**	**10 (5.2)**

*p<0.05

Among the neoplasms of the participants in Group A, breast cancer prevailed in 31 (32.3%), followed by digestive system cancer in 17 (17.7%), cancer of male genital organs in 10 (10.4%) and lymphoma in 10 (10.4%), while 29.2% correspond to other types of neoplasms.

In Table 2, the medians (25-75 percentile) of the CRE-Breve and QoL are displayed with the respective domains in both groups. The significant use of negative RSC is observed in Group A, as well as lower scores in the physical and psychological wellbeing domains of quality of life.

**Table 2:** Distribution of median scores (25-75 percentile) on the CRE-Breve*, quality of life score on the McGill Quality of Life Questionnaire and its domains between the groups studied (n=192). Botucatu, SP, Brazil, 2015

		**Group**					**p**
		**A**			**B**		
		**Median**	**p 25-75**		**Median**	**p 25-75**	
CRE-Breve*							
	**Positive**	**3.01**	**2.67-3.51**		**3.11**	**2.44-3.47**	**0.688**
	**Negative**	**1.53**	**1.28-1.66**		**1.40**	**1.20-1.60**	**0.010**
	**Total**	**3.74**	**3.56-3.98**		**3.79**	**3.52-4.03**	**0.583**
**Quality of life**							
	**Part A**	**8.00**	**7.00-10.00**		**8.00**	**7.00-9.00**	**0.470**
	**Physical wellbeing**	**7.00**	**6.00-8.00**		**8.00**	**6.25-10.00**	**0.011**
	**Psychological wellbeing**	**5.12**	**3.50-8.00**		**6.75**	**4.50-8.62**	**0.025**
	**Existential wellbeing**	**8.83**	**8.00-9.45**		**8.33**	**7.33-9.33**	**0.109**
	**Support**	**9.00**	**8.00-10.00**		**8.00**	**7.00-9.00**	**0.000**
	**Physical symptoms**	**8.83**	**6.66-10.00**		**9.33**	**6.08-10.00**	**0.607**
	**Total**	**7.64**	**6.78-8.55**		**7.90**	**6.55-8.86**	**0.649**

*CRE-Breve: Brief Religious-Spiritual Coping

In the bivariate analysis, a weak but significant correlation was found between the RSC and QoL scores in both groups assessed: Group A rho=0.32, p<0.01; Group B rho=0.24, p=0.02.

**Figure 1:**
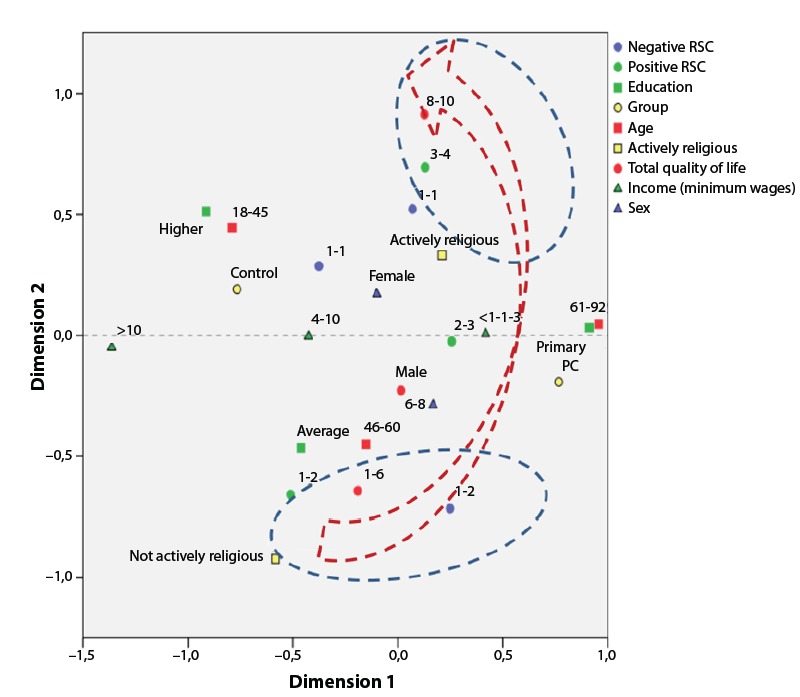
Multiple correspondence analysis diagram among CRE-Breve scores, quality of life and other covariables in the research. Botucatu, SP, Brazil, 2015

In Figure 1, the correspondences are described between the quality of life scores in Groups A and B and the following variables: sex, family income, education, age, RSC (positive and negative) and being actively religious. The exploratory correspondence analysis consisted of two dimensions, responsible for explaining between 26 and 18% of the data set, and the QoL scores were mainly located in dimension 2. A direct correspondence was observed between the QoL scores and positive RSC, negative RSC and being actively religious (arrows pointing in the same direction).

In Table 3, the multivariate analyses applied are displayed, using the generalized linear model for quality of life and CRE-Breve. The variables male sex, Catholic religion and total CRE-Breve presented a significant association to explain the variation in the QoL scores.

**Table 3:** β coefficients of generalized linear model for the McGill Quality of Life Questionnaire and CRE-Breve* scores (n=192). Botucatu, SP, Brazil, 2015

	**Quality of life**			CRE-Breve*	
	**β coefficient**	**p**		**β coefficient**	**p**
**Group A**	**-0.177**	**0.493**		**0.148**	**0.005**
**Male sex**	**0.699**	**0.001**		**-0.101**	**0.200**
**Age**	**0.001**	**0.919**		**0.045**	**0.953**
**Education (primary vs. higher)**	**-0.567**	**0.138**		**0.055**	**0.346**
**Income (lowest vs. highest quartile)**	**-0.769**	**0.611**			
**With partner**	**0.143**	**0.550**		**-0.046**	**0.335**
**Catholic religion**	**0.492**	**0.026**		**-0.032**	**0.470**
**Actively religious**	**-0.116**	**0.641**			
**Kinship of resident (direct relative vs. alone)**	**-0.618**	**0.184**			
**Total quality of life**	**-**	**-**		**0.0470**	**0.000**
Total CRE-Breve*	**1.448**	**0.001**			

*CRE-Breve: Brief Religious-Spiritual Coping.

What the RSC is concerned, a positive association was identified between the participants in Group A (p=0.005) and the total quality of life (p=0.000).

In Table 4, the factors are presented that interfered in the perceived quality of life in the past two days. As observed, the disease symptoms and fear of death stand out among the factors that most strongly influenced the perception of the construct.

**Table 4:** Answers related to part A of the McGill Quality of Life Questionnaire. Factors that interfered in the perceived quality of life in the past two days. Botucatu, SP, Brazil, 2015

**Factors**	N (%)*
**Kindness and support from the family**	**28 (29.1)**
**Pain**	**15 (15.6)**
**Fear of dying**	**10 (10.4)**
**Not being able to sleep, eat and pray**	**9 (9.3)**
**Solitude and distancing from relatives and friends**	**7 (7.3)**
**Symptoms of disease treatment/hair loss**	**7 (7.3)**
**Financial difficulties**	**5 (5.2)**
**Did not answer**	**4 (4.1)**
**Death of a loved one**	**4 (4.1)**
**Being able to pray and go to church**	**3 (3.1)**
**Lack of understanding of the family about the disease**	**2 (2.0)**
**Need to do a new surgery**	**2 (2.0)**
**Go out and do shopping**	**1 (1.0)**
**Improvement of pain and symptoms**	**1 (1.0)**
**Anxiety about test results**	**1 (1.0)**
**Total**	**102.5**

*The results add up to more than 100% because more than one category was identified in the answers to this question.

## Discussion

The limits of the study results refer to its cross-sectional design, as holding interviews at a single moment may not be enough to picture the magnitude of changes that can occur in the palliative care phase.

On the other hand, quality of life assessment has been used as an indicator to guide care practices and support the definition of public health policy strategies. Nevertheless, there is a lack of studies that assess quality of life in palliative care in Brazil, despite the relevance of the theme.

What the quality of life score is concerned, little difference was observed in relation to the groups that considered it to be relatively good. Quality of life assessment has been acknowledged as a complex task due to the abstract and subjective nature of the concept, for which no consensus definition exists yet. The World Health Organization (WHO) quality of life definition itself is complex and demonstrates positive and negative facets, besides the multiple dimensions of the concept in coping with the inter-relation between the environment and individual physiopathological aspects, independence level, social relationships and personal beliefs^17^.

In addition, it should be kept in mind that this inter-relation exists within a certain cultural context, within the context of a value system, which individuals live in, and in relation to their objectives, concerns, expectations and standards. Therefore, any quality of life measure needs to reach exactly this set of elements within an index or score that reflects the perception of different individuals in different circumstances of life^18^.

In fact, in a prospective study, in which the quality of life of 105 cancer patients attended at a tertiary hospital outpatient clinic was assessed, impaired global wellbeing and a low general quality of life were revealed^19^.

In a recent systematic review on the theme, it was suggested that a wide range of quality of life domains should be considered in the assessment of terminal patients in palliative care. The authors concluded that measures need to be refined to identify issues the patients value, such as the preparation for death and aspects inherent in health care provision, among others, which the instruments available in the literature often do not address^20^. In addition, it is known that the determining factors of cancer patients’ quality of life often are not well understood^21^.

In this research, a statistically significant difference was observed in relation to the domains physical wellbeing, psychological wellbeing and support when the groups were compared. This finding is supported by a study involving lung cancer patients, in which the quality of life was lower than in the general population, being affected by the severity of the disease and the number of symptoms. In that study, fatigue and respiratory problems contributed to reduce the psychological dimension of quality of life^22^.

In a study involving 158 advanced cancer patients, it was shown that high levels of hopelessness, impaired body image and emotional suffering were the main factors associated with psychological stress^23^.

To reach the second specific objective in this study, multiple linear regression analysis was applied to the quality of life score, with some explanatory variables. The quality of life presented a statistically significant positive association with the male sex, Catholic religion and total CRE-Breve. These data reveal the beneficial effect of religion on the perceived quality of life of these patients at such a difficult moment.

What the RSC is concerned, the results demonstrated the participants’ high usage level of this strategy, mainly the positive factor, in both groups. Nevertheless, a statistically significant difference was observed in terms of negative RSC when the groups were compared. This result is probably due to the negative emotional impact of cancer which, in turn, affects the cancer patients’ religion/spirituality. The uncertainty about the future and the hopelessness that mark these people’s life at that moment probably affected the use of RSC.

A positive association between RSC and total quality of life was identified among the participants in Group B. This fact can be due to the diverging moment of life and the healthy participants’ health condition can justify the results found, even if little is known on the relations between RSC and quality of life of incurable cancer patients in the literature.

In another study, involving 350 terminal patients, mostly married women with lung cancer, it was shown that the patients use a range of coping strategies. The use of emotional support and acceptance strategies was correlated with a better quality of life in that research^24^.

The limits of the research results initially refer to the application of the questionnaire at a single moment, which may not be enough to picture the range of interferences and difficulties the patient experiences in that period. In addition, the lack of studies on quality of life and RSC of palliative cancer care patients made it difficult to compare the results, but also showed that other studies are needed in the area. Hence, future studies with longitudinal designs are proposed to guide nursing actions for these clients, in view of associations between sex, religion and the use of RSC.

## Conclusion

These study results indicate that the participants’ quality of life was relatively good, and that the psychological domain was the most affected in Group A. When associated with sociodemographic and clinical variables, male participants who were actively religious and obtained higher RSC scores revealed a better perception of this construct.

The use of RSC was high and the use of positive coping prevailed. Nevertheless, when the groups were compared, the palliative care patients made greater use of the negative factor. In this research, healthy participants with better quality of life scores showed better religious-spiritual coping.
